# Establishment of an NPK nutrient monitor system in yield-graded cotton petioles under drip irrigation

**DOI:** 10.1186/s13007-023-01068-0

**Published:** 2023-09-04

**Authors:** Zhiqiang Dong, Yang Liu, Minghua Li, Baoxia Ci, Xiaokang Feng, Shuai Wen, Xi Lu, Zheng He, Fuyu Ma

**Affiliations:** 1 School of Agriculture, Shihezi University, Shihezi, 832003 Xinjiang People’s Republic of China; 2 Guiyang Healthcare Vocational University, Guiyang, 550081 Guizhou People’s Republic of China; 3 National and Local Joint Engineering Research Center of Information Management and Application Technology for Modern Agricultural Production (XPCC), Shihezi, 832003 Xinjiang People’s Republic of China

**Keywords:** Petiole, Cotton, Nutritional monitor system, Back propagation neural network, Algorithm optimization

## Abstract

**Background:**

The determination of nutrient content in the petiole is one of the important methods for achieving cotton fertilization management. The establishment of a monitoring system for the nutrient content of cotton petioles during important growth periods under drip irrigation is of great significance for achieving precise fertilization and environmental protection.

**Methods:**

A total of 100 cotton fields with an annual yield of 4500–7500 kg/ha were selected among the main cotton-growing areas of Northern Xinjiang. The nitrate nitrogen (NO_3_^−^–N), inorganic phosphorus (PO_4_^3−^–P) and inorganic potassium (K^+^–K) content and yield of cotton petioles were recorded. Based on a yield of 6000 kg/ha as the dividing line, a two-level and yield-graded monitoring system for NO_3_^−^–N, PO_4_^3−^–P and K^+^–K in cotton petioles during important growth periods was established, and predictive yield models for NO_3_^−^–N, PO_4_^3−^–P and K^+^–K in petioles during important growth periods were established.

**Results:**

The results showed found that the yields of the 100 cotton fields surveyed were normally distributed. Therefore, two yield grades were classified using 6000 kg/ha as a criterion. Under different yield-graded, the NO_3_^−^–N, PO_4_^3−^–P and K^+^–K content of petiole at important growth stages was significantly positively correlated with yield. Further, the variation range of NO_3_^−^–N, PO_4_^3−^–P and K^+^–K content in petioles could be used as a standard for yield-graded. In addition, a yield prediction model for the NO_3_^−^–N, PO_4_^3−^–P and K^+^–K content of petioles was developed. The SSO-BP validation model performed the best (R^2^ = 0.96, RMSE = 0.06 t/ha, MAE = 0.05 t/ha) in the full bud stage, which was 12.9% higher than the BP validation model. However, the RMSE and MAE were decreased by 86.7% and 88.1%, respectively.

**Conclusion:**

The establishment of NPK nutrition monitor system of cotton petioles under drip irrigation based on yield-graded provides an important basis for nutrition monitor of cotton petiole under drip irrigation in Xinjiang. It also provides a new method for cotton yield prediction.

## Background

Optimal application amount and timing of fertilizers containing nitrogen (N), phosphorus (P) and potassium (K) are important in determining cotton growth, yield, economic benefit, and reducing environmental pollution [1, 2]. Therefore, rapid and accurate estimation of the N, P and K content in cotton is the basis of fertilizer application, and an important indicator of the nutritional level of cotton [3].

The levels of nitrate nitrogen (NO_3_^−^–N), inorganic phosphorus (PO_4_^3−^–P) and inorganic potassium (K^+^–K) in cotton plants reflect the nutritional status. Different parts of the cotton plant have different levels well of NO_3_^−^–N, PO_4_^3−^–P and K^+^–K contents in the order petioles > stem > leaves [4]. The petiole is generally used for nutritional monitor in cotton plants [5, 6]. The determination of petiole NO_3_^−^–N, PO_4_^3−^–P and K^+^–K content [7, 8] in cotton is a rapid, simple, and accurate method and is widely used in cotton growing regions. A nutritional diagnostic tool for determining NO_3_^−^–N, PO_4_^3−^–P and K^+^–K content in cotton petioles in lacking in Xinjiang. Previous studies by Chinese scholars [9, 10] focused on plot trials at one location. Therefore, there is an urgent need to develop a novel tool that can be used for nutritional monitor in cotton petioles, thus realizing sustainable development of the cotton industry [11]. Currently, there is a lack of field rapid nutrition monitoring technology system for drip irrigation cotton nutrition management in Xinjiang. However, previous scholars' monitoring indicators for NO_3_^−^–N, PO_4_^3−^–P and K^+^–K content in drip irrigation cotton petioles are relatively outdated and need to be updated and corrected.

The level of NO_3_^−^–N, PO_4_^3−^–P and K^+^–K content of the petiole at different fertility periods reflects the yield potential of cotton [12]. Cotton yield provides a theoretical basis for cotton management [13]. The content of NO_3_^−^–N, PO_4_^3−^–P, and K^+^–K in cotton petiole correlates with yield. Studies have shown that the content of NO_3_^−^–N in petiole was significantly correlated with the cotton yield at bud stage, early flowering stage, full flowering stage and boll stage, which could be a sensitive indicator of N nutrition status in cotton [14, 15]. Wang et al. [16] conducted a study on the effect of different nitrogen fertilizer dosages on NO_3_^−^–N concentration in the last four leaf petioles of cotton using a reflectometer to diagnose nitrogen nutrition indicators and recommend top dressing. The results showed that there was a highly significant correlation between NO_3_^−^–N concentration in cotton plant petioles and yield during flowering stage, flower and boll stage and boll stage.

Cotton yield can be predicted by the NO_3_^−^–N, PO_4_^3−^–P and K^+^–K content in petioles during important growth stages [17]. Wei et al. [9] have shown that the NO_3_^−^–N content in the last four leaf petioles of cotton during the bud stage is significantly correlated with yield, which can sensitively indicate the N nutrition status of cotton and serve as a recommended diagnostic period for cotton topdressing.The accuracy of cotton yield prediction is directly related to the fertilization level during important growth periods [19]. Therefore, reliable prediction methods [18] can help excessive fertilizer application, reduce environmental pollution and unnecessary costs [20]. With the rapid rise of artificial intelligence, neural network technology [21] provides new methods and approaches for agricultural applications. Neural network technology can handle multidimensional data and provides strong support for improving yield prediction models [22]. The back propagation neural network (BP) is widely used in cotton production [23]. However, BP has several limitations and shortcomings [24], it is highly sensitive to the initial weight and easily converges to the local minimum. The introduction of an optimization [25] algorithm can effectively improve the global search ability and convergence speed of BP and reduce the possibility of falling into local optimum.

This study selected 100 cotton fields in northern Xinjiang with annual yields ranging from 4500 to 7500 kg/ha, and used rapid cotton petiole nutrition monitoring instruments for nutrient monitoring. The feasibility of establishing a yield-graded drip irrigation cotton petiole nutrition monitoring system and applying petiole nutrition for yield prediction model construction was explored. The NPK nutrient diagnostic system for cotton petioles in Northern Xinjiang was improved as well as providing a new method for yield prediction.

## Methods

### Study area and field experiment

**Fig. 1:**
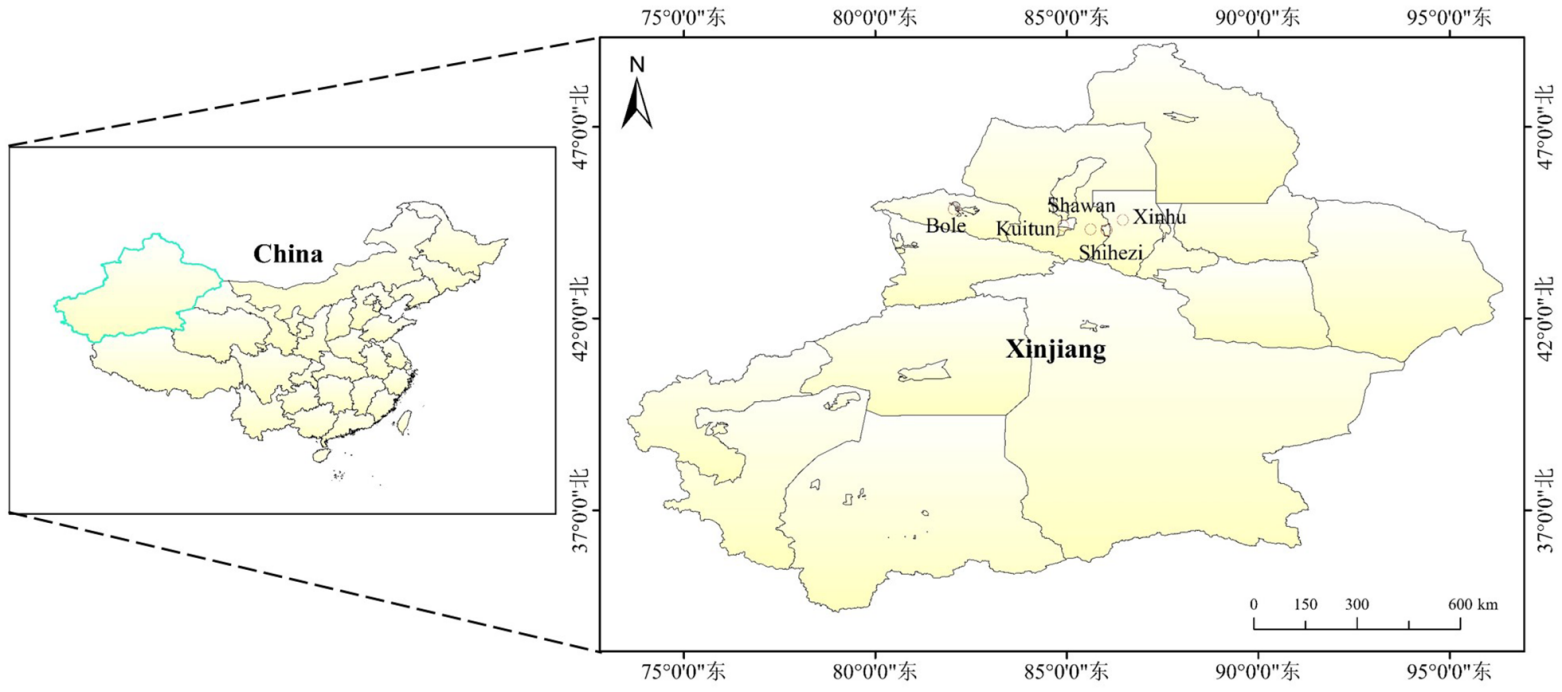
Study area location

A total of 100 cotton fields with annual yield level of 4500–7500 kg/ha were selected for sampling test, including 30 cotton fields selected in 2019 and 70 cotton fields in 2020. The cotton fields (Fig. 1) were located in Shihezi city of the eighth division of Xinjiang Production and Construction Corps (85°94^′^E, 44°27^′^N), Shawan city of Tacheng Prefecture (85°56^′^E, 44°29^′^N), Kuitun city of Yili Kazak Autonomous Prefecture (84°89^′^E, 44°45^′^N), Xinhu farm of the sixth division of Xinjiang Production and Construction Corps (86°22^′^E, 44°28^′^N), and Bole City of Bortala Mongolian Autonomous Prefecture of Xinjiang Uygur Autonomous Region (82°1^′^E, 44°93^′^N).

The cotton fields were located in the main cotton-growing areas in the northern region of Xinjiang. Xinjiang has a temperate continental arid and semi-arid climate. The total cumulative annual temperature above 10 °C is 3000–4000 °C, the average rainfall is above 150–200 mm, and the annual frost-free period is about 140–185 days.

Cotton was planted in machine harvesting mode: 1 film, 3 tubes, and 6 rows, with a plant spacing of about 9.5–10 cm. Cotton was sown using mechanical planters in early to mid-April and was picked mechanically in early October. Fertilizer application rates were 270–300 kg/ha N, 90–120 kg/ha P_2_O_5_, and 80–100 kg/ha K_2_O, respectively. The fertilizers were applied using drip irrigation technology.

### Petioles collection

Sampling was carried out on sunny mornings between 10:00 and 12:00 am. During this period, the metabolism of cotton was expected to be in a state of dynamic balance. In addition, the NO_3_^−^–N, PO_4_^3−^–P, and K^+^–K stored in the cotton plant could best reflect the relative relationship between nutrient absorption and assimilation.

Each cotton field was divided into 3.33hm as the measurement area of petiole NO_3_^−^–N, PO_4_^3−^–P, and K^+^–K, with a total area of about 333.33 hm. Ten samples were obtained at the full bud stage, full bloom stage, full boll stage of the cotton plant. The samples were obtained from each cotton field using the ‘S’ shaped collection mode. Five cotton plant leaves with petioles were collected from the last four leaves (10 days before topping) and the last two leaves (10 days after topping) at each sampling point. Therefore, a total of 50 leaves were collected.

### Determination of NO_3_^−^–N, PO_4_^3−^–P and K^+^–K and in petioles

**Fig. 2:**
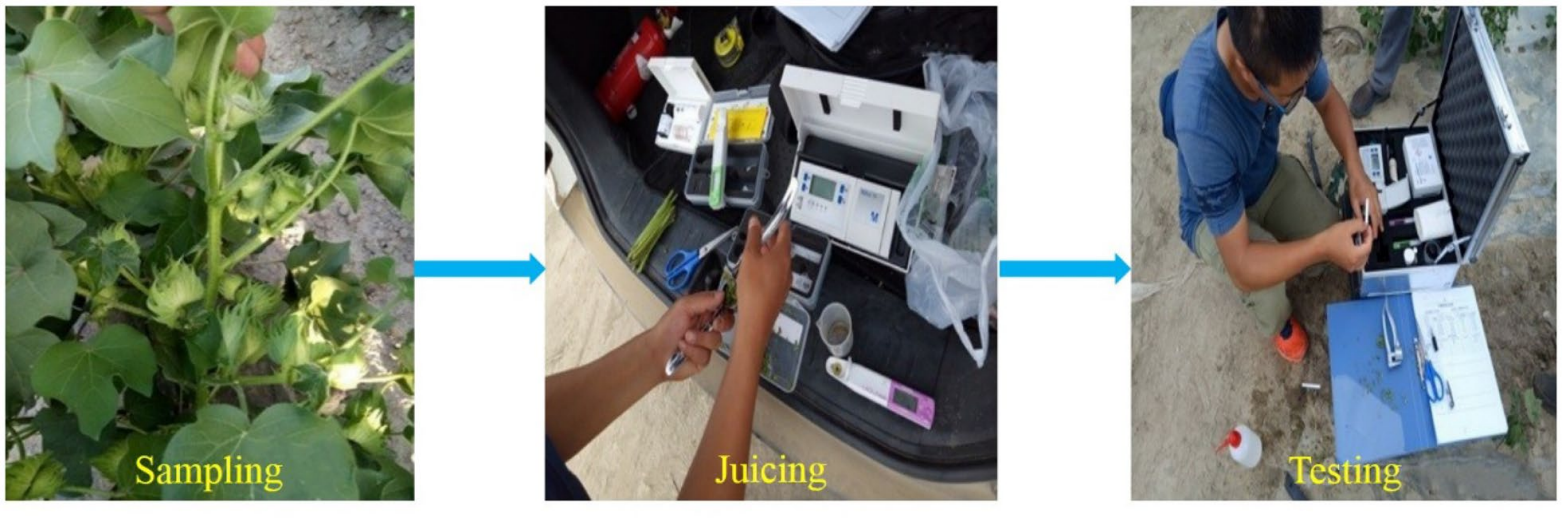
Petioles NO_3_^−^-N, PO_4_^3−^-P and K^+^-K test procedure

The samples were washed with distilled water, and the petioles and leaves were separated. The petioles were cut and pressed. The contents of NO_3_^−^**–**N and K^+^**–**K in cotton petioles were determined using LAQUA Twin NO_3_^−^ meter and K^+^ meter (HORIBA Inc., Japan), while the PO_4_^3−^–P content was determined using RQflex20 Reflectoquant (Merck Inc., Germany). A detailed description of the instruments and the procedures are shown in Table 1 and Fig. 2.

**Table 1:** LAQUA twin NO_3_^−^, K^+^and RQflex20 PO_4_^3−^ instrument profile

			
Instruments name	LAQUA twin NO_3_^−^	LAQUA twin K^+^	RQflex20 PO_4_^3−^
Measuring principles	Ion electrode	Ion electrode	Reflected light
Volume of samples required	0.3–2.0 mL	0.3–2.0 mL	0.3–2.0 mL
Scope of measurement	2–9900 mg L^−1^	2–9900 mg L^−1^	5–120 mg L^−1^

### Yield determination

Three sampling points were selected during the boll opening stage from a 3-m-long plot. The number of harvested plants, boll weight, and the calculated yield were recorded. The average value of the three samples was considered as the final yield.

### Statistical analysis

Statistical analysis was conducted to analyze the petiole NO_3_^−^–N, PO_4_^3−^–P and K^+^–K content and yield data during three important growth stages in 100 cotton fields. Yield equal to 6000 kg/ha was considered the cut-off value for statistical analysis. A total of 30 samples were valid for yield between 4800 and 6000 kg/ha, and 270 samples were valid for petiole determination of NO_3_^−^–N, PO_4_^3−^–P and K^+^–K content. For yields between 6000 and 7100 kg/ha, there were 50 valid samples for yield and 450 valid samples for determination of petiole NO_3_^−^–N, PO_4_^3−^–P and K^+^–K content. The remaining 20 abnormal yield samples and 180 abnormal petiole NO_3_^−^–N, PO_4_^3−^–P and K^+^–K content samples were not included in the statistical analysis. The anomalous samples were the points where the petiole NO_3_^−^–N, PO_4_^3−^–P and K^+^–K deviated from the yield fit to a large extent. All data were analyzed using SPSS software version 20.0 (SPSS Inc, Chicaho, Illimois, USA).

### Modeling methods

The BP [26] is a multilayer feed-forward network trained by error back propagation algorithm. It is one of the most widely used neural network models. Its learning rule is to use the steepest descent method to continuously adjust the weights and thresholds of the network through back propagation, thus minimizing the sum of squared errors of the network. The BP is created in MATLAB r2019b software. The BP adopts a three-layer structure, the hidden layer node is 10, the number of iterations is 200, and the learning rate is 0.01.

**Fig. 3:**
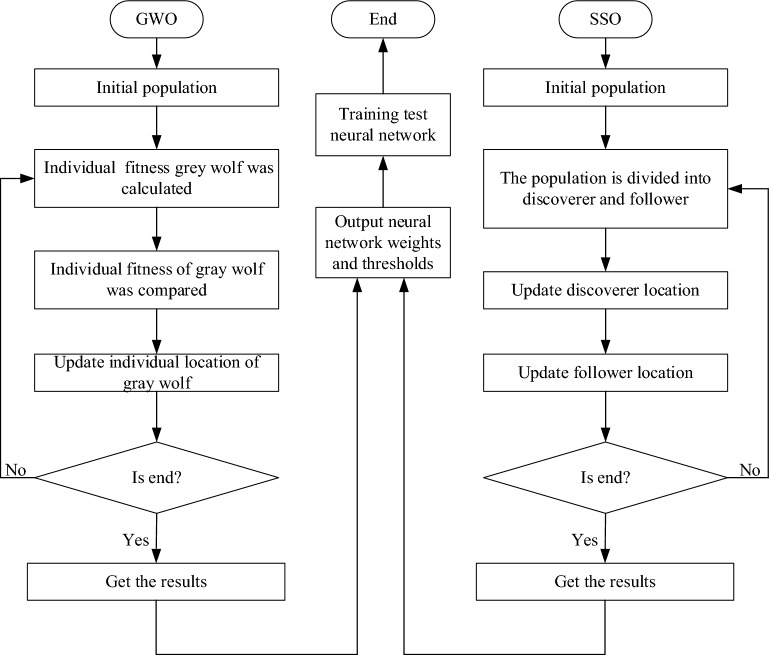
Optimizing BP flow chart

The sparrow searches optimizer (SSO) algorithm [27] is based on the swarm intelligence optimization algorithm which is based on the sparrow foraging and avoiding the predator behavior. At the same time, a certain proportion of individuals in the population are selected for detection and early warning. If danger is found, they will give up food and safety first. The sparrow foraging optimization method is used to improve the speed and ability of global optimization search. It avoids the drawbacks of the prediction value of original BP falling into local optimum. The sparrow search optimizer backpropagation neural network (SSO-BP) is created in MATLAB r2019b software (MathWorks, Inc. Natick, Massachusetts, USA). The number of SSO-BP population is 20, the hidden layer node is 10, and the number of iterations is 20. The process is shown in Fig. 3.

The grey wolf optimizer (GWO) algorithm [28] is a population intelligent optimization algorithm based on the inspiration of grey wolf's preying activities. It has the characteristics of strong convergence, few parameters and easy implementation. Aiming at the problems of low precision and slow convergence of original BP, gray wolf predator–prey optimization method is used to improve the speed and ability of global optimization search. It avoids the drawback of the prediction value of original BP falling into local optimum. The grey wolf optimizer backpropagation neural network (GWO-BP) is created in MATLAB r2019b software. The number of populations of GWO-BP is 20, the number of hidden layer nodes is 10, and the number of iterations is 20. The process is shown in Fig. 3.

The independent verification data awere from the experimental cotton field of Shihezi University teaching experimental field. The fertilization rates were 300 kg/ha N, 109.8 kg/ha P_2_O_5_ and 91.8 kg/ha K_2_O.

The content of NO_3_^−^–N, PO_4_^3−^–P and K^+^–K in petiole were used as independent variables to model yield. Further, independent validation samples were used to test the model. The coefficient of determination (R^2^) Eq. 1, root mean square error (RMSE) Eq. 2 and mean absolute error (MAE) Eq. 3 were used to test the accuracy of the model and to determine the best prediction model.

1 $$R^{2} \, = \,1 - \frac{{\mathop \sum \nolimits_{i = 1}^{n} \left( {F_{i} - T_{i} } \right)^{2} }}{{\mathop \sum \nolimits_{i = 1}^{n} \left( {T_{i} - \overline{{T_{i} }} } \right)^{2} }}$$

2 $$RMSE\, = \,\sqrt {\frac{1}{n} \times \mathop \sum \limits_{i = 1}^{n} \left( {F_{i} - T_{i} } \right)^{2} }$$

3 $$MAE\, = \,\frac{1}{n}\mathop \sum \limits_{i = 1}^{n} \left| {F_{i} - T_{i} } \right|$$

In the formula: Fi and Ti are predicted and true values, while n is the number of samples.

## Results

### Descriptive statistical characteristics of major nutrients based on yield-graded petioles

**Fig. 4:**
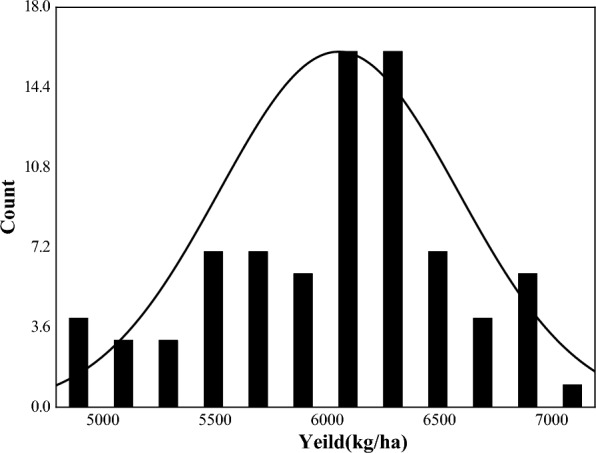
Normal distribution of yield

The effective yield samples (Table 2), and yield accords with the normal distribution are shown in Fig. 4. The maximum value of the samples with yield less than 6000 kg/ha was 5963.11 kg/ha, the minimum value was 4833.89 kg/ha, and the average value was 5493.09 kg/ha. The samples with a yield of over 6000 kg/ha, had a maximum value of 7012.88 kg/ha, minimum value of 6026.13 kg/ha, and an average value of 6397.26 kg/ha. The coefficient of variation for yield less than 6000 kg/ha was higher than that of yield more than 6000 kg/ha.

**Table 2:** Descriptive statistical characteristics of yield

Element	> 6000 kg/ha					< 6000 kg/ha				
	Max	Min	AV	SD	CV%	Max	Min	AV	SD	CV%
Yield (kg/ha)	7012.88	6026.13	6397. 26	281.74	4.4	5963.11	4833.89	5493.09	331.86	6.0

Max, Min, AV, SD and CV represent the maximum, minimum, average, standard deviation and coefficient of variation, respectively

Under the yield level of 4800–6000 kg/ha (Table 3), the NO_3_^−^–N content in petiole decreased gradually during growth stage. However, the highest NO_3_^−^–N content was observed during full bud stage, while the lowest was in the full boll stage. The average contents of NO_3_^−^–N in petioles at full bud stage, full bloom stage, full boll stage were 8393, 6327, and 4703 mg/L, respectively. The content of PO_4_^3−^–P in petiole increased first and then decreased during the growth stage, with the highest content observed during full flowering stage and the lowest content was observed during full bud stage. The average content of PO_4_^3−^–P in petiole was 152, 261 and 163 mg/L at full bud stage, full bloom stage, full boll stage, respectively. The trend of K^+^-K content in petiole was consistent with that of NO_3_^−^–N content in petiole. The average contents of K^+^–K in petioles were 8200, 6213 and 3253 mg/L, respectively. In full bud stage, the variation coefficient of PO_4_^3−^–P content in petiole was the highest, and that of K^+^-K content in petiole was the lowest.

**Table 3:** Descriptive statistical characteristics of petiole NO_3_^−^-N, PO_4_^3−^-P and K^+^-K at yield level of 4800–6000 kg/ha

Element	Full bud stage					Full bloom stage					Full boll stage				
	Max	Min	AV	SD	CV%	Max	Min	AV	SD	CV%	Max	Min	AV	SD	CV%
NO_3_^—^N (mg/L)	9500	7000	8393	736.69	8.8	7000	5000	6327	597.66	9.5	5500	3000	4703	710.26	15.1
PO_4_^3—^P (mg/L)	180	85	152	28.96	19.1	300	200	261	29.48	11.3	200	100	163	30.24	18.6
K^+^-K (mg/L)	9000	7000	8200	624.22	7.6	7000	5000	6213	597.54	9.6	4000	2000	3253	599.27	18.4

Max, Min, AV, SD and CV represent the maximum, minimum, average, standard deviation and coefficient of variation, respectively

Under the yield level of 6000–7100 kg/ha (Table 4), the changes of NO_3_^−^–N, PO_4_^3−^–P and K^+^-K in petioles with growth stage were consistent with the yield level of 4800–6000 kg/ha. The average content of NO_3_^−^–N in petiole was 9798, 7640, and 6556 mg/L at full bud stage, full bloom stage, full boll stage, respectively. The average content of PO_4_^3−^–P in petiole was 204, 348, and 252 mg/L at full bud stage, full bloom stage, full boll stage, respectively. The average contents of K^+^-K in petioles were 9230, 7574, and 5580 mg/L at full bud stage, full bloom stage, full boll stage, respectively.

**Table 4:** Descriptive statistical characteristics of petiole NO_3_^−^-N, PO_4_^3−^-P and K^+^-K at yield level of 6000–7100 kg/ha

Element	Full bud stage					Full bloom stage					Full boll stage				
	Max	Min	AV	SD	CV%	Max	Min	AV	SD	CV%	Max	Min	AV	SD	CV%
NO_3_^—^N (mg/L)	11000	9000	9798	608.94	6.2	8500	6500	7640	705.95	9.2	7500	5000	6556	842.29	12.9
PO_4_^3—^P (mg/L)	265	100	204	50.65	24.8	400	270	348	43.98	12.6	300	185	252	38.63	15.3
K^+^-K (mg/L)	10000	8000	9230	578.26	6.3	8500	6000	7574	813.36	10.7	6500	4000	5580	725.34	13.0

Max, Min, AV, SD and CV represent the maximum, minimum, average, standard deviation and coefficient of variation, respectively

### Nutritional monitor of NPK in yield-graded petioles

**Fig. 5:**
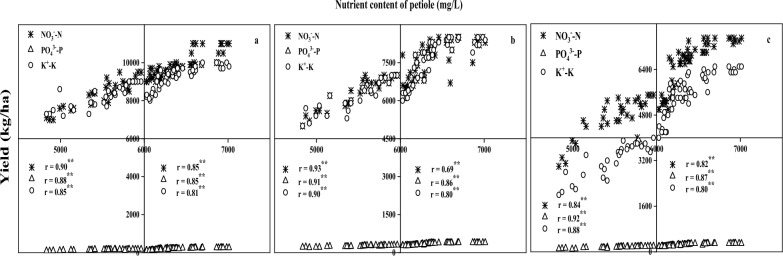
Correlation between NO_3_^−^-N, PO_4_^3−^-P and K^+^-K content in petiole and yield at main growth stage. **a** full bud stage. **b** full bloom stage. **c** full boll stage. ^**^ Correlation is significant at the 0.01 level

There was a significant positive correlation between petiole NO_3_^−^–N content and yield at each growth stage (Fig. 5a). At the full flowering stage, under the yield level of 4800–6000 kg/ha, the content of NO_3_^−^–N in petiole had the highest significance with yield (r = 0.93). In contrast, at the full bud stage, the NO_3_^−^–N content in petiole had the highest significance with yield (r = 0.85) at the yield level of 6000–7100 kg/ha.

The content of PO_4_^3−^–P in petiole also showed a significant positive correlation (Fig. 5b). At the  full boll stage, the yield was 4800–6000 kg/ha, the petiole PO_4_^3−^-P content and yield had the highest significance (r = 0.92). Under the yield of 6000–7100 kg/ha, the content of PO_4_^3−^–P in petiole was the highest (r = 0.87).

The relationship between petiole K^+^–K content and NO_3_^−^–N and PO_4_^3−^–P content was the same (Fig. 5c). At the full flowering stage, the yield was 4800–6000 kg/ha, and the petiole K^+^–K content and yield had the highest significance (r = 0.90). At the full bud stage, the significant correlation between petiole K^+^–K content and yield was the highest at the yield level of 6000–7100 kg/ha (r = 0.81).

According to the content characteristics of NO_3_^−^–N, PO_4_^3−^–P and K^+^–K in cotton petioles under drip irrigation and the relationship between them and yield. The nutritional monitor of NPK in yield-graded petioles during important growth periods was established, as shown in Table 5.

**Table 5:** Monitor system for NO_3_^−^-N, PO_4_^3−^-P and K^+^-K of petiole in yield-graded

Growth period									Yield(kg/ha)
Full bud stage			Full bloom stage			Full boll stage			
NO_3_^—^N (mg/L)	PO_4_^3—^P (mg/L)	K^+^-K (mg/L)	NO_3_^—^N (mg/L)	PO_4_^3—^P (mg/L)	K^+^-K (mg/L)	NO_3_^—^N (mg/L)	PO_4_^3—^P (mg/L)	K^+^-K (mg/L)	
9000–11000	100–265	8000–10000	6500–8500	270–400	6000–8500	5000–7500	185–300	4000–6500	6000–7100
7000–9500	85–180	7000–9000	5000–7000	200–300	5000–7000	3000–5500	100–200	2000–4000	4800–6000

### Establishment and verification of a yield prediction model

The prediction models during important growth periods based on SSO-BP, GWO-BP, and BP were established. As shown in Table 6, the modeling accuracy of GWO-BP was significantly higher than that of SSO-BP and BP. The GWO-BP performed the best (R^2^ = 0.96, RMSE = 0.11t/ha, MAE = 0.08 t/ha). It was 3.2% higher than the SSO-BP. The RMSE and MAE were decreased by 35.3% and 33.3%, respectively. However, it was 9.1% higher than the BP. The RMSE and MAE were decreased by 42.1% and 42.9%, respectively.

**Table 6:** Yield modeling and verification of different methods

Growth period	Methods	Modeling				Validation			
		Number of samples	R^2^	RMSE (t/ha)	MAE (t/ha)	Number of samples	R^2^	RMSE (t/ha^2^)	MAE (t/ha)
Full bud stage	SSO-BP	80	0.85	0.21	0.16	20	0.96	0.06	0.05
	GWO-BP	80	0.95	0.12	0.10	20	0.95	0.09	0.08
	BP	80	0.82	0.23	0.18	20	0.85	0.45	0.42
Full bloom stage	SSO-BP	80	0.85	0.20	0.17	20	0.95	0.07	0.06
	GWO-BP	80	0.91	0.16	0.13	20	0.91	0.10	0.08
	BP	80	0.83	0.22	0.17	20	0.83	0.12	0.11
Full boll stage	SSO-BP	80	0.93	0.17	0.12	20	0.91	0.09	0.07
	GWO-BP	80	0.96	0.11	0.08	20	0.90	0.10	0.08
	BP	80	0.88	0.19	0.14	20	0.88	0.17	0.15

Independent test data were used for verification. As shown in Table 6, the SSO-BP had the highest stability which was close to the GWO-BP. Among them, the SSO-BP performed the best (R^2^ = 0.96, RMSE = 0.06 t/ha, MAE = 0.05 t/ha) in the full bud stage, which was 12.9% higher than the BP. However, the RMSE and MAE were decreased by 86.7% and 88.1%, respectively.

## Discussion

The yield was used as the evaluation standard to establish a new petiole NPK nutrition monitor system. A total of 100 cotton fields with an annual yield of 4500–7500 kg/ha in the main cotton-growing areas of Northern Xinjiang were selected for the study. Firstly, the yield was positively distributed and was found to be in accordance with the law of positive distribution. We also found that the NO_3_^−^–N, PO_4_^3−^–P and K^+^–K content of petiole correlated with yield during important growth periods, consistent with previous studies [9, 12, 13]. Therefore, the yield was used as an evaluation standard to determine the NO_3_^−^–N, PO_4_^3−^–P and K^+^–K content monitor system of petiole in two grades. Previous studies used plot experiments with different NPK content, on a single test site. However, the studies also used yield as the main evaluation standard to determine the NO_3_^−^–N, PO_4_^3−^–P and K^+^–K content of petiole [7, 10]. This study solved the pain point that was limited to a single community. The entire experimental data was sourced from real cotton fields in northern Xinjiang, and the latest monitoring standard range of NO_3_^−^–N, PO_4_^3−^–P and K^+^–K content in cotton petiole during important growth periods was corrected. The establishment of this system provides an important basis for the management of cotton fertilization under drip irrigation in Xinjiang, reducing fertilizer waste and protecting the environment. Therefore, the results are highly applicable and can be used as reference values for NPK nutrition monitor in cotton petioles under drip irrigation in Northern Xinjiang.

Several studies have focused on yield predictions during crop growth [30]. Many studies used hyperspectral and unmanned aerial vehicle equipment to estimate cotton yield [31, 32]. These studies established a relationship between the spectral parameters of the equipment and yield. Measurements from these devices allow for a rapid inversion of cotton yield [35]. However, due to differences in ecological and environmental conditions, irrigation and fertilization management, it was difficult to determine the cotton yield using these inversion indicators accurately. Some scholars have used statistical dynamics growth simulation models [33] to predict yield. Kem et al. [34] constructed a multiple linear regression model, using meteorological data and soil moisture content from meteorological reanalysis as the prediction factor for the model. The most suitable model was selected using stepwise linear regression method, providing a simple equation with good explanatory coefficients that can accurately estimate crop yield. But using growth models only focuses on the internal influencing factors of cotton yield, neglecting external influencing factors, and can only predict the yield around two months before harvest. In this study, we combined the results of previous studies and selected a representative cotton growing area in Northern Xinjiang, applied the petiole NPK monitoring instrument to measure the petiole NO_3_^−^–N, PO_4_^3−^–P and K^+^–K content of drip irrigation cotton. A drip irrigation cotton yield prediction model was construed using different neural network methods and petiole NO_3_^−^–N, PO_4_^3−^–P and K^+^–K content. The fitting effect of the model based on the NO_3_^−^–N, PO_4_^3−^–P, and K^+^–K content of petiole was excellent. In addition, the model had high stability and strong prediction ability. This shows that the accuracy and universality of using petiole major nutrient content to predict cotton yield in Northern Xinjiang was high.

**Fig. 6:**
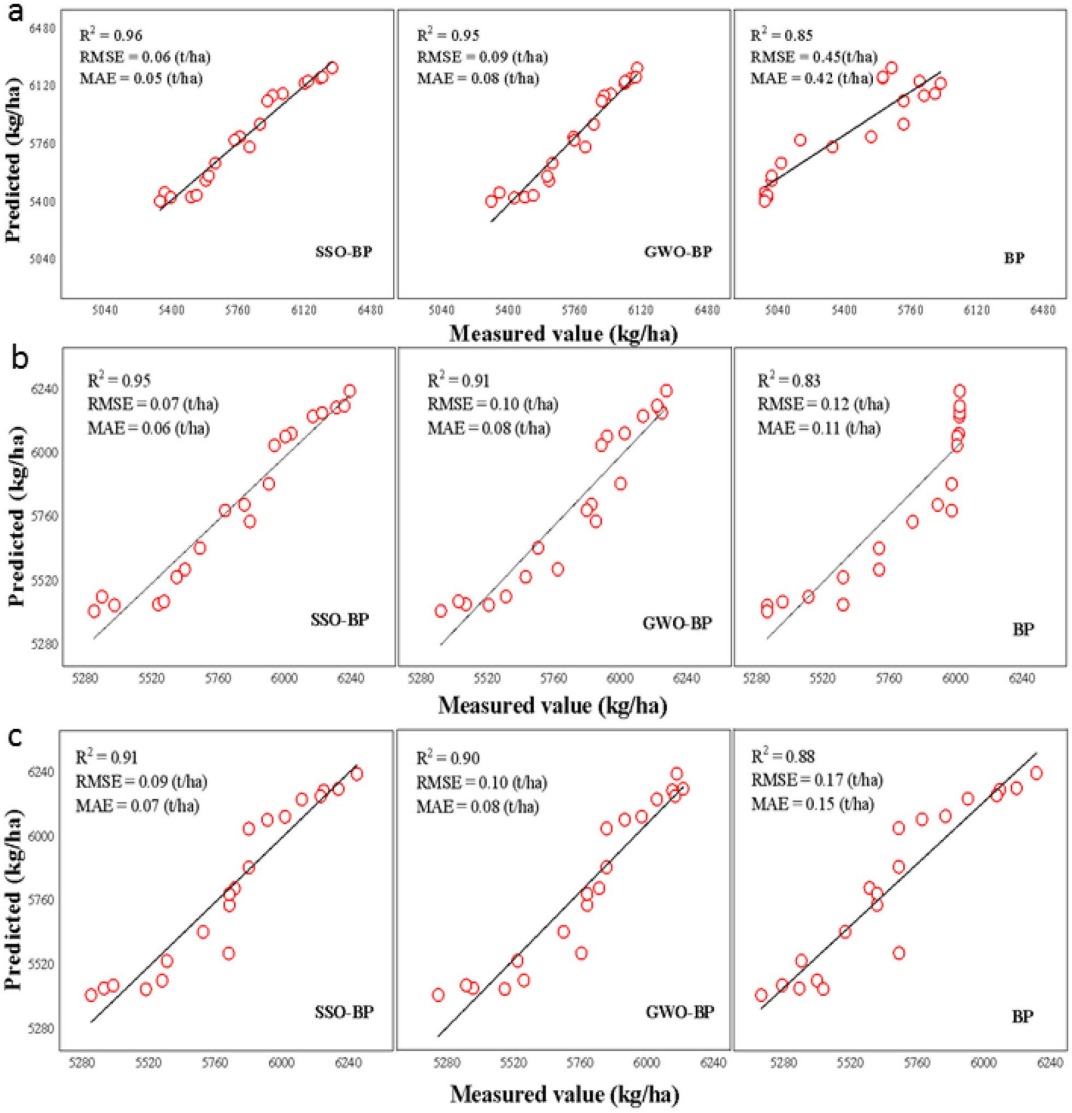
Yield verification. **a**, Validation model of full bud stage. **b**, Validation model of full bloom stage. **c**, Validation model of full boll stage

The BP [28, 29] is one of the most widely used neural networks. However, it is very sensitive to the initial weight, has relatively slow convergence, and is sometimes affected by the over fitting phenomenon [25]. we used the SSO [36] and GWO [37, 38] to optimize the initial weight and threshold of BP. The optimized BP had better modeling and prediction ability than BP as shown in Fig. 6. The SSO algorithm [39] is an intelligent optimization algorithm proposed in 2020. In this study, the SSO algorithm was used to optimize the BP neural network to verify the good performance of the model and further prove the application potential of the SSO algorithm in agricultural production.

## Conclusions

The yield had a significant positive correlation with petiole NO_3_^−^–N, PO_4_^3−^–P, and K^+^–K content. Therefore, we determined a yield-graded  drip irrigation cotton petiole NPK  monitor system. The prediction of cotton yield based on the NO_3_^−^–N, PO_4_^3−^–P, and K^+^–K content of petiole had high stability and accuracy. In addition, the optimized BP had a substantial improvement in predictive ability over the traditional BP. This study provides an important reference standard for the monitor of NPK in  drip irrigation cotton petioles in Xinjiang. Further, this study offers new insights into the application of the SSO algorithm in agriculture.

## Data Availability

The remotely sensed and field sampling data used in this study is available from the corresponding author on reasonable request.
